# Evaluation of vardenafil for the treatment of subjective tinnitus: a controlled pilot study

**DOI:** 10.1186/1477-5751-8-3

**Published:** 2009-02-17

**Authors:** Birgit Mazurek, Heidemarie Haupt, Agnieszka J Szczepek, Jörg Sandmann, Johann Gross, Burghard F Klapp, Holger Kiesewetter, Ulrich Kalus, Timo Stöver, Philipp P Caffier

**Affiliations:** 1 Department of Otorhinolaryngology, Tinnitus Centre and Molecular Biology Research Laboratory, Charité – Universitätsmedizin Berlin, Charitéplatz 1, 10117 Berlin, Germany; 2 Department of Internal Medicine, Division Psychosomatics and Psychotherapy, Charité – Universitätsmedizin Berlin, Charitéplatz 1, 10117 Berlin, Germany; 3 Institute for Transfusion Medicine, Charité – Univeritätsmedizin Berlin, Charitéplatz 1, 10117 Berlin, Germany; 4 Department of Otorhinolaryngology, Medical University of Hannover, Carl-Neuberg-Strasse 1, 30625 Hannover, Germany

## Abstract

**Background:**

Vardenafil (Levitra^®^) represents a potent and highly selective phosphodiesterase type 5 (PDE5) inhibitor, which is established for treatment of various diseases. There are several unpublished reports from patients stating that vardenafil has a considerable therapeutic effect on their concomitant tinnitus. This pilot study was conducted to specifically assess the effect of vardenafil in patients with chronic tinnitus.

**Methods:**

This trial was based on a prospective, randomized, double-blind, placebo-controlled, parallel group design. Fourty-two consecutive subjects with mon- or binaural chronic tinnitus received 10 mg vardenafil (N = 21) or matching placebo tablets (N = 21) administered orally twice a day over a period of 12 weeks. Clinical examination and data acquisition took place at each visit: at baseline, after 4 weeks, after 12 weeks (end of treatment with study medication), and at non-medicated follow-up after 16 weeks. Assessment of clinical effectiveness was based on a standardized tinnitus questionnaire (TQ), the Short Form 36 health survey (SF-36), audiometric measurements (mode, pitch and loudness of tinnitus; auditory thresholds) and biomarkers of oxidative stress in patients' blood (malondialdehyde, protein carbonyl, homocysteine and total antioxidative status). Therapeutic efficacy was evaluated by comparison of subjective and objective parameters with baseline data between both treatment groups (ANCOVA).

**Results:**

Vardenafil had no superior efficacy over placebo in the treatment of chronic tinnitus during this study. The primary efficacy criterion 'TQ total score' failed to demonstrate significant improvement compared to placebo. Subjective reports of TQ subscales and general quality of life areas (SF-36), objective audiometric examinations as well as investigated biomarkers for oxidative stress did not reveal any significant treatment effects. The safety profile was favorable and consistent with that in other vardenafil studies.

**Conclusion:**

Although hypoxia and ischemia play a special role in the pathogenesis of tinnitus, the PDE5-inhibitor-induced increase of nitric oxide-mediated vasodilatation exerted no specific influence on tinnitus symptomatology. Considering the unclear risk of rarely associated hearing impairment, systemic application of vardenafil or other PDE5 inhibitors prove to be not appropriate for therapy of chronic tinnitus.

## Background

Tinnitus is defined as subjective perception of noises without existence of an objective sound source [1]. Chronic tinnitus is an etiologically inconsistent, nosologically complex symptom lasting for more than 3 months. Tinnitus-related distress, decompensation and resulting secondary symptomatology represent a worldwide major health care problem with an enormous social and economic demand for therapeutic treatment [2]. According to epidemiological investigations, more than 37 million Americans experience tinnitus, about 20% of them have prolonged tinnitus requiring clinical intervention [3,4]. Patients with hearing impairment suffer significantly more often from tinnitus than others; conversely, approx. 90% of all tinnitus patients are affected by impaired hearing [5,6].

Noise (especially impulse noise), stress, ototoxic substances, sudden hearing loss as well as head and neck injuries are factors that may contribute to the pathogenesis of tinnitus [7-9]. Exposure to intense noise leads to a decrease of oxygen partial pressure and blood flow in the cochlea [10-12]. Ischemia and hypoxia are known to greatly affect the function of cochlea [13,14]. Hence, adequate microcirculation is essential to ensure optimal function of the inner ear. The blood flow into the cochlea is derived from the basilar artery *via *anterior inferior cerebellar artery (AICA), labyrinthine artery and finally *via *functional end arteries. The intra-cochlear diffusion distances are relatively long. Except for the basal part of cochlea, the main blood supply comes from spiral modiolar artery (SMA). As pointed out by Tange [15], clinical features vary according to the site of arterial obstruction. Interruption of SMA leads to hair cell loss, but does not affect the stria vascularis (SV). Circulation disorders in SV cause general degeneration, but the sensory cells remain intact. Occlusion of labyrinthine artery leads to almost complete degeneration of the inner ear [16,17]. Ischemic conditions impact the expression of a number of hypoxia inducible factor 1 (HIF-1)-dependent genes, which are involved in acute as well as chronic changes in circulation and in the signal transduction [18,19]. Nitric oxide (NO), a known mediator of vasodilatation and neurotransmission, is present in various parts of the cochlea [20]. NO is the major endothelium-derived relaxing factor that interacts with soluble guanylate cyclase generating cyclic guanosine monophosphate (cGMP). cGMP activates protein kinases and leads to relaxation of surrounding smooth muscle, resulting in vasodilatation and increase in blood flow [21,22].

Phosphodiesterase type 5 (PDE5) inhibitors are used for the treatment of various cardiovascular, pulmonary, neurological and urogenital diseases [23-25]. Vardenafil (Levitra^®^) represents a potent and highly selective PDE5 inhibitor, which is established for therapy of erectile dysfunction (ED) and pulmonary arterial hypertension [26-28]. There are several unpublished anecdotal reports from male patients suffering from ED and concomitant tinnitus, stating that vardenafil has a considerable therapeutic effect on their tinnitus. PDE5 inhibitors block the degradation of cGMP by PDE5 [29]. As for NO-dependent mechanisms, increased cGMP levels were shown to act on pericytes, which ultimately leads to vasodilatation in the SV improving blood supply to hair cells [30,31]. This could constitute a mechanism contributing to the hypothesized effect of PDE5 inhibitors on tinnitus.

Considering the causative role, which hypoxia/ischemia and oxidative stress may play in the pathogenesis of tinnitus, the question aroused whether PDE5-inhibitor-induced increase of cGMP and potentiated NO-dependent vasodilatation exert a defined and specific influence on tinnitus symptomatology. Therefore, we designed and conducted this pilot study to specifically assess the effect of vardenafil in patients with chronic tinnitus. The goal was to evaluate therapeutic tinnitus improvement and to show superior efficacy of vardenafil over placebo by means of defined subjective and objective variables. Concerning the latter, emphasis was placed on validated blood parameters serving as biomarkers of oxidative stress, namely malondialdehyde (MDA, indicator of lipid oxidation [32]), protein carbonyl (PC, marker of protein oxidation [33]), the total antioxidative status (TAS [34]), and homocysteine (HCY, biomarker associated with vascular disease [35]).

## Methods

### Study design and procedure

This phase II clinical study was based on a prospective, randomized, double-blind, placebo-controlled, parallel group design meeting good clinical practice criteria. The study was conducted as an adaptive design with an interim analysis after termination of 2 × 20 subjects. The trial was conducted in accordance with the Declaration of Helsinki and upon approval by the local ethical review board. Before the study was started, all patients underwent a baseline evaluation including detailed medical history, otorhinolaryngologic examination, full neurootological diagnostics, electrocardiogram (ECG) and blood tests. Within 1 week after screening visit V1, eligible tinnitus patients were randomly assigned (by means of computer-generated block randomization) to either vardenafil or placebo group at randomization visit V2 (baseline). Subjects received treatment with study medication over a period of 12 weeks, i.e., from V2 to end of treatment visit V4. Subsequently, they went into a non-medicated follow-up for another 4 weeks until final follow-up visit V5 after 16 weeks.

Study treatment consisted of 10 mg vardenafil tablets or matching placebo tablets administered twice daily (bid) orally. Study medication was supplied at V2 and after 4 weeks at interim visit V3 in carded blister pack dispensers containing medications for 4 and 8 weeks respectively. Tablets were to be swallowed with a glass of water (approx. 200 ml) in the morning and evening, with a dosing interval of approx. 12 hours. Subjects had to return the whole medication packaging including empty blisters and unused medication. The number of tablets returned was counted in order to check compliance.

Clinical examination and data acquisition took place at each visit. Subjective questionnaires concerning tinnitus and quality of life were administered at baseline (V2), during treatment (V3), at the end of treatment (V4), and 4 weeks after treatment with study medication (V5). Audiometric measurements along with assessment of biomarkers of oxidative stress and vascular disease were also performed from V2 to V5. Furthermore, patients were told to immediately stop medication and show up for premature discontinuation (PD) visit in case of experienced intolerance or adverse events (AEs) related to the study medication.

### Patients

Study participants were recruited from the patients of Tinnitus Center at the Charité Department of Otorhinolaryngology (Oct 2006 to May 2007). A consecutive sample of 51 subjects with mon- or binaural chronic tinnitus was enrolled in the screening process. Main inclusion criteria were: male and female patients, age >18 to <65 years, tinnitus duration >3 months, chronic subjective cochlear tinnitus, no treatment of tinnitus within 4 weeks prior to study entry, available linguistic and intellectual skills to fill out the questionnaires.

Exclusion criteria comprised particular otologic findings (acute or intermittent tinnitus, history of Menière's disease, tumors of the middle ear/inner ear/cerebellopontine angle), specific diseases (history of malignancies, multiple sclerosis, retinal disorders, liver/hematological/cardiovascular diseases), pregnancy or breast-feeding women, abnormal laboratory values (e.g., creatinine clearance <30 ml/min, elevation of aspartate aminotransferase (AST) or alanine aminotransferase (ALT) ≥ 3-fold upper limit of normal), previous use of PDE5 inhibitors, special concomitant medication (nitrates or NO donors, alpha1-adrenoceptor antagonists, potent inhibitors of cytochrome P- 450 3A4), and any other concurrent treatment of tinnitus during the trial (pharmacological and non-pharmacological).

Trial data were analyzed using three populations: the safety population, the intent-to-treat (ITT) population and the per-protocol (PP) population. The safety population was defined as all patients who received at least one dose of randomized study medication and had any post-randomization safety data. The ITT population included all randomized patients who received at least one dose of study medication and had at least one post-baseline assessment in any clinical variable (psychometric data, audiometric measurements). The PP population was defined as a subgroup of the ITT population who completed the study as scheduled and for whom no major protocol violations were observed (e.g., poor compliance, insufficient drug exposure, prohibited concomitant medication).

**Figure 1:**
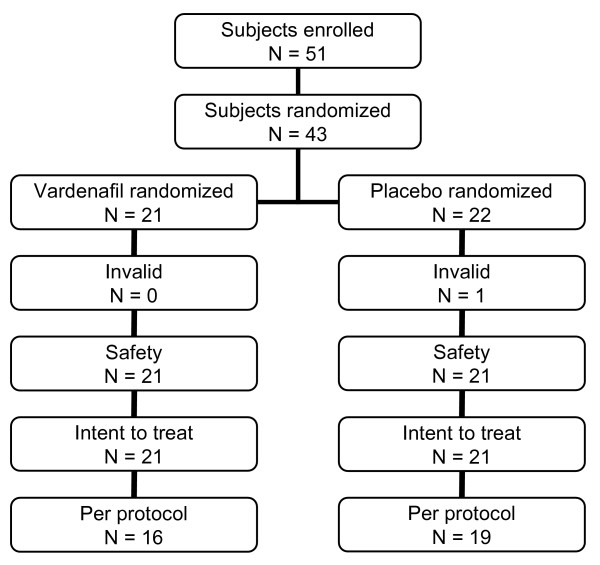
**Flow chart of study population**.

After their written informed consent, 43 patients were included in the study and randomized: 21 to vardenafil and 22 to placebo group. Since one subject randomized to placebo rejected participation, both the safety and the ITT population finally included 21 patients of each treatment group. The PP population consisted of 16 subjects receiving vardenafil and 19 subjects receiving placebo. A flow chart of our study population is illustrated in Figure 1.

### Subjective instruments for examination

Psychometric data were acquired by two established self-evaluation instruments: a standardized tinnitus questionnaire (TQ) and the Short Form 36 health survey (SF-36). We used TQ by Goebel and Hiller [36] to register tinnitus-associated psychological and psychosocial complaints. TQ by Goebel and Hiller is based on Hallam's TQ [37] and it was specifically developed and validated for the German population. Using TQ enables measuring the tinnitus impairment with six partially correlating factors based on constructs of information-processing such as irrational concepts, over-generalization and attitudes of helplessness. Patients had to indicate how much each of the 52 statements corresponded to their actual state on a 3-step category rating scale (true, partly true, not true). The TQ total score as well as specific fields of distress were assessed by means of subscales labeled: emotional distress, cognitive distress, intrusiveness, auditory perceptual difficulties, sleep disturbances, and somatic complaints. The resulting TQ total score (0–84 points) allows a subdividing into four severity levels: low (1–30), moderate (31–46), severe (47–59) and very severe impairment (60–84 points). Tinnitus is considered to be 'compensated' at a TQ level of = 46 (no secondary symptoms) and 'decompensated' at a TQ level of = 47 (permanent annoyance and psychological strain; accompanied by complaints like depression, anxiety, impaired sleep and concentration). The applied TQ has been sufficiently evaluated and is regarded to be the best available method in Germany at present to determine the tinnitus severity level [36].

The SF-36 questionnaire was applied as a well-established assessment tool for general health care settings as well as accepted methodology for the observation of clinical course and therapy control in tinnitus [38,39]. The German edition used was validated for German conditions [40]. The SF-36 is interpreted as a health-related quality-of-life (QoL) measurement with 36 category rating scaled items, 8 scales summarizing subsets of these items and 2 summary measures (physical health, mental health). Scores range from 0 to 100, with higher scores indicating higher levels of functioning and associated QoL. Patients were asked to rate the level of their present health condition. In order to provide a basis for the assessment of achieved results, the scores were compared with mean values and standard deviations of a German normal sample.

### Objective Parameters

Safety and tolerability of study medication was assessed by vital signs (heart rate, blood pressure), 12-lead ECG, and the following blood parameters: ALT, AST, creatinine, glucose, creatine kinase (CK), creatine kinase muscle-brain (CK-MB), potassium, and human chorionic gonadotropine (hCG). Safety analyses were performed on the safety population. Treatment groups were compared with respect to incidence rates of premature termination, treatment-emergent adverse events (AEs), and abnormalities of ECG and blood parameters.

Audiometric measurements were conducted to characterize the tinnitus and to investigate auditory thresholds at tone exposures with varying frequencies from 0.125 kHz to 10 kHz (pure tone audiogram). Tinnitus examination comprised the determination of mode, pitch and loudness of tinnitus. These individual tinnitus characteristics were detected *via *a headset earphone device asking the subject to indicate matching. Tinnitus loudness was assessed by comparing the perceived tinnitus to the known loudness of a defined acoustic stimulus presented to the contralateral ear. The intensity of this comparative tone or noise was identified, and the loudness was interpreted as the threshold in terms of dB at which the tinnitus is detected [41]. Tympanometric examinations were performed to preclude pathological functioning in the tympanum and the auditory meatus.

In addition, biomarkers were determined for objective pharmacodynamic evaluation of oxidative stress and disease progression during therapy [35,42]. Therefore, the levels of MDA, PC, TAS and HCY were measured in the patients' blood. The plasma (EDTA) concentration of MDA was determined by high performance liquid chromatography (HPLC) using a kit of fluorometric detection supplied by Chromsystems (Munich, Germany). Serum concentration of the PC was measured using the ZenTech immuno-assay kit (Zenith Technology, Dunedin, New Zealand). The TAS was determined in plasma (lithium heparin) using the Randox assay (Crumlin, Antrim, UK). The level of HCY was analyzed in serum using HPLC with fluorometric detection after reduction and derivatization (Recipe, Munich, Germany). The plasma/serum component was separated (centrifugation at 3000 rpm for 10 min, within 30 min), and 0.5 ml aliquots were stored at -20°C until analysis.

### Statistical Analysis

Means ± standard deviations (SD) were calculated for all parameters measured. The primary efficacy analysis was based on the TQ score in the ITT sample. Inferential statistics utilized the last observation carried forward (LOCF) value, i.e., the score observed at the last visit under treatment (or PD). Statistical analysis was based on an analysis of covariance (ANCOVA) with baseline as covariate and the LOCF value as dependent variable. Factor was 'treatment'. The homogeneity of regression slopes was tested. Analysis was repeated for the PP population. The secondary efficacy and outcome variables were as follows: QoL (SF-36), audiometric data, blood parameters of oxidative stress, safety and tolerability parameters. These variables were also analyzed via descriptive statistics and the ANCOVA model. It was decided to determine the study with conclusion of efficacy when the actual p-value was ≤ 0.0233. If the p-value exceeded 0.5, no efficacy conclusion would be drawn from the data and the study would stop. P-values of >0.0233 – 0.5 were considered to plan a second study.

## Results

### Baseline patient characteristics

Relevant general and otologic characteristics of the patients are presented in Table 1. Patients assigned to both treatment groups were comparable with regard to baseline characteristics including race (all Caucasian), sex-ratio, age, socio-demographic criteria, weight and body-mass-index (BMI). Regarding risk factors and concomitant diseases (High Level Terms, MedDRA version 10.0), vardenafil and control group showed similar smoking and drinking habits, 14 subjects had systemic arterial hypertension and 8 were diagnosed with psychiatric disorders (depression, anxiety). Audiometric parameters revealed no significant differences concerning tinnitus variables and degree of tinnitus annoyance. Altogether, 17 subjects suffered from a unilateral tinnitus, 25 from a bilateral one. Tinnitus was compensated in 27 patients (cT) with low to moderate impairment (mean TQ score = 22.5), and decompensated in 15 patients (dT) with severe to very severe impairment (mean TQ score = 60). The pre-therapeutic course of tinnitus was constant in 71% and progressive in 29% of study participants. The mean pre-existing tinnitus duration was 6 years (range, 8 months to 28 years). A concomitant hearing loss was prevalent in about 90% of all patients. Normacusis was not seen in verum group, but in 4 placebo subjects (8 tinnitus ears).

**Table 1:** Major baseline characteristics.

**Characteristics**	**All patients** **(N = 42)**	**Vardenafil** **(N = 21)**	**Placebo** **(N = 21)**
Gender			
Male	30 (71%)	14 (67%)	16 (76%)
Female	12 (29%)	7 (33%)	5 (24%)
Age, years (mean ± SD)	49.0 ± 10.2	51.3 ± 10.0	46.7 ± 10.2
Body weight, kg (mean ± SD)	81.6 ± 17.0	82.6 ± 19.2	80.6 ± 14.8
BMI, kg/m^2 ^(mean ± SD)	26.5 ± 3.9	27.1 ± 4.2	25.9 ± 3.5
Non-smokers	27 (64%)	16 (76%)	11 (52%)
Smokers	15 (36%)	5 (24%)	10 (48%)
Pack-year history^a ^(mean ± SD)	17.3 ± 10.8	19.9 ± 10.7	15.9 ± 11.3
Current alcohol consumption			
Abstinent	15 (36%)	8 (38%)	7 (33%)
Light/moderate (≤ 2 units^b ^per day)	27 (64%)	13 (62%)	14 (67%)
Tinnitus duration, years (mean ± SD)	6 ± 5	7 ± 6	5 ± 3
Course of tinnitus			
Constant	30 (71%)	17 (81%)	13 (62%)
Progressive	12 (29%)	4 (19%)	8 (38%)
Tinnitus prevalence			
Ears without tinnitus	17 (20%)	9 (21%)	8 (19%)
Ears with tinnitus	67 (80%)	33 (79%)	34 (81%)
Tonal type	60 (90%)	29 (88%)	30 (88%)
Noise type	7 (10%)	4 (12%)	4 (12%)
Hearing level in tinnitus ears^c^			
Normacusis (<20 dB)	8 (12%)	0 (0%)	8 (23%)
Mild hearing loss (20–40 dB)	49 (73%)	28 (85%)	21 (62%)
Moderate hearing loss (41–60 dB)	9 (14%)	4 (12%)	5 (15%)
Severe hearing loss (>60 dB)	1 (1%)	1 (3%)	0 (0%)
Tinnitus annoyance			
cT (TQ total score ≤ 46)	27 (64%)	13 (62%)	14 (67%)
dT (TQ total score ≥ 47)	15 (36%)	8 (38%)	7 (33%)

^a^(number of years smoking) × (number of cigarettes per day)/20; ^b^One unit = one glass of wine or beer; ^c^amount of mean hearing loss as detected in pure tone audiogram (1, 2, 3, 4 kHz); cT – compensated tinnitus, dT – decompensated tinnitus, TQ – tinnitus questionnaire.

Data are expressed as number and percentage of subjects, unless otherwise noted.

### Treatment compliance and safety analysis

Study medication was taken for 77 ± 21 days on average (range 3–90 days, median 84 days = 12 weeks) without major differences between treatment duration in the vardenafil and placebo group (72 ± 26 vs. 81 ± 13 days). Pill count revealed that the average number of doses taken by all subjects was 148 ± 44 doses (range 4–175 doses, median 167 doses = 2 × 1 tablet per day). Eighty-one percent of all patients had a study drug intake compliance rate of 80 to 100%; 90.5% of placebo and 85.8% of verum group subjects were compliant within the tolerated range (80–120%). Descriptive data of adherence and compliance with study medication showed no discernible differences between active treatment and placebo group.

Concerning safety, there were no clinically important differences between treatment groups for vital signs or laboratory parameters at any time point. Mean baseline values of blood pressure and pulse did not change under therapy. ECG findings, cardiac measures and all other parameters investigated in this study raised no clinical concerns.

Distribution of AEs (Preferred Terms, MedDRA version 10.0) and drop-outs are listed in Table 2. Treatment-emergent AEs were reported by 38% of vardenafil and 14% of placebo patients. The most common complaints were headache, diarrhea, and nasal congestion. 28.5% of vardenafil and 9.5% of placebo patients experienced non-serious AEs considered drug-related, which led to PD in 4 vardenafil subjects (1 woman with diarrhea, 1 woman with intolerable heat sensation, 1 man with prolonged penile erection, and 1 man with flushed face and nasal mucosal swelling) and in 1 placebo subject (1 man with dizziness, headache, and nausea). In all patients with PD due to medication, the experienced symptoms resolved within one week after discontinuation of the study drug. Serious or fatal AEs were not observed.

**Table 2:** Incidence rates of adverse events (AEs) and premature discontinuation.

	**Vardenafil** **(N = 21)**	**Placebo** **(N = 21)**
Treatment-emergent AEs	8 (38.1%)	3 (14.3%)
Drug-related AEs	6 (28.5%)	2 (9.5%)
- Headache	1 (4.75%)	2 (9.5%)
- Diarrhea	2 (9.5%)	0 (0%)
- Nasal congestion	2 (9.5%)	0 (0%)
- Prolonged penile erection	1 (4.75%)	0 (0%)
Serious or fatal AEs	0 (0%)	0 (0%)
Premature discontinuation	5 (23.8%)	2 (9.5%)
- due to drug-related AEs	4 (19.05%)	1 (4.75%)
- due to poor compliance	1 (4.75%)	1 (4.75%)

Data are expressed as number and percentage of subjects in both treatment groups.

### Efficacy analysis

#### TQ

**Figure 2:**
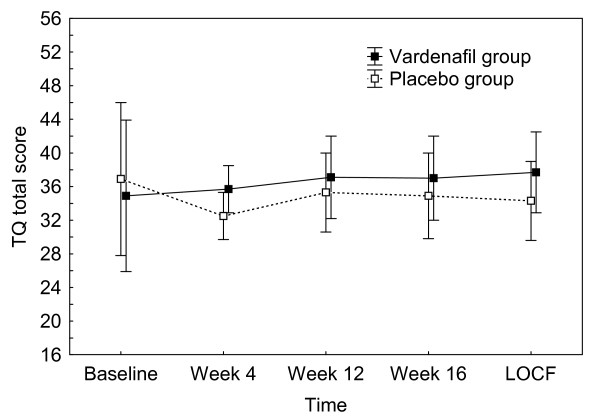
**Time course of total TQ scores from baseline to week 16 with LOCF evaluated in vardenafil and placebo groups**. Given are the LS-means and 95% confidence intervals of the ITT population.

The initial mean TQ scores were at the same level in verum and control group (34.9 and 36.9). After 16 weeks (LOCF), placebo-treated patients reported a slight improvement by 1.7 points, but vardenafil-treated subjects showed a mean deterioration by 2 points on average (Table 3). However, within- and between-groups differences were clinically not relevant. The course of TQ total scores (LS-means) from V2 to V5 with LOCF in both treatment groups is shown in Figure 2. The analysis of ITT sample did not demonstrate any treatment effects. The analysis of the PP sample with 16 verum and 19 placebo subjects yielded consistent results. Effects remained the same when reference was made to the 12-week active treatment period at V4, when subjects were still under drug exposure.

**Table 3:** TQ total score with subscores (mean ± SD) at baseline and at week 16 (LOCF) including ANCOVA results from baseline to week 16 (LOCF) of ITT samples.

	**Vardenafil (N = 21)**			**Placebo (N = 21)**			
TQ	Baseline	LOCF	Changes	Baseline	LOCF	Changes	p-value^a^

**Total score**	34.9 ± 20.0	36.9 ± 21.3	2.0 ± 7.9	36.9 ± 21.0	35.2 ± 22.4	-1.7 ± 12.4	0.29
**Subscores** Emotional distress	9.1 ± 5.8	9.7 ± 6.5	0.6 ± 3.3	10.0 ± 7.1	9.3 ± 7.2	-0.7 ± 4.0	0.29
Cognitive distress	6.6 ± 4.6	6.6 ± 4.8	0 ± 1.8	7.0 ± 5.2	6.4 ± 5.5	-0.6 ± 3.3	0.52
Intrusiveness of tinnitus	9.8 ± 3.8	9.9 ± 3.6	0.1 ± 2.2	9.4 ± 3.9	8.9 ± 4.3	-0.5 ± 2.9	0.34
Aud. perc. difficulties^b^	4.6 ± 4.5	5.7 ± 4.1	1.1 ± 2.4	5.3 ± 3.4	5.5 ± 4.1	0.2 ± 2.7	0.26
Sleep disturbances	3.0 ± 2.6	3.0 ± 2.8	0 ± 1.2	3.2 ± 2.8	3.3 ± 3.0	0.1 ± 1.0	0.88
Somatic complaints	1.9 ± 2.1	2.0 ± 2.3	0.1 ± 1.0	1.9 ± 1.7	1.9 ± 1.8	0 ± 1.5	0.81

^a^F-Test, ANCOVA; ^b^Auditory perceptual difficulties

In addition, the groups were subdivided into patients with cT (14 placebo and 13 vardenafil subjects) and dT (7 placebo and 8 vardenafil subjects) according to their different degree of tinnitus severity. The ANCOVA did not reveal any significant effects of vardenafil on cT or dT patients (data not shown).

#### TQ subscales

With regard to TQ subscales, none of the various domains of tinnitus-associated complaints underwent substantial changes during therapy (Table 3). While all TQ sub-scores showed slight improvements or maintained stable under placebo treatment, there was a tendency for minor deteriorations under vardenafil medication, especially for the subscales emotional distress and auditory perceptual difficulties. However, all differences in changes from baseline were statistically not significant.

#### SF-36

The general health status SF-36 did not show any significant changes (Table 4). Comparison of average baseline values with the German norm values indicated a relatively reduced condition with regard to emotional and social competence as well as problems in fulfilling the own expectations (role – physical). The ANCOVA results did not reveal any treatment effects on one of the QoL areas.

**Table 4:** SF-36 subsets (mean ± SD) at baseline and at week 16 (LOCF) including ANCOVA results from baseline to week 16 (LOCF) of ITT samples.

**SF-36 subsets**	**Vardenafil (N = 21)**			**Placebo (N = 21)**			
	Baseline	LOCF	Changes	Baseline	LOCF	Changes	p-value^a^

Physical funct.^b^ (85.7 ± 22.1)	80.5 ± 19.9	74.3 ± 28.0	-6.3 ± 15.0	77.0 ± 26.9	77.0 ± 27.6	0.0 ± 14.0	0.18
Role-physical (83.7 ± 31.7)	58.8 ± 41.6	55.0 ± 44.9	-3.8 ± 40.0	63.1 ± 44.4	51.2 ± 47.1	-12 ± 33.2	0.53
Bodily pain (79.1 ± 27.4)	60.1 ± 29.9	51.3 ± 31.4	-8.8 ± 17.4	65.2 ± 28.4	62.9 ± 30.4	-2.3 ± 19.9	0.21
General health (68.1 ± 20.2)	59.5 ± 22.8	55.6 ± 25.4	-3.8 ± 10.9	49.1 ± 20.3	48.0 ± 22.4	-1.2 ± 12.0	0.52
Vitality (63.3 ± 18.5)	55.3 ± 21.6	52.3 ± 22.6	-3.0 ± 12.1	43.8 ± 20.1	42.5 ± 25.3	-1.2 ± 13.9	0.75
Social funct.^b^ (88.8 ± 18.4)	73.2 ± 22.1	71.4 ± 24.4	-1.8 ± 9.9	62.5 ± 32.8	64.9 ± 33.0	2.4 ± 24.2	0.70
Role-emotional (90.4 ± 25.6)	63.1 ± 42.9	61.4 ± 46.2	-1.7 ± 47.8	60.4 ± 43.0	54.0 ± 44.1	-6.4 ± 41.8	0.64
Mental health (73.9 ± 16.4)	67.0 ± 19.2	61.6 ± 21.6	-5.4 ± 13.6	52.0 ± 20.4	51.0 ± 22.9	-1.1 ± 14.3	0.59

^a^F-Test, ANCOVA; ^b^functioning.

In the left column, average German norms (± SD) are given in brackets.

#### Audiometric data

The audiometric examinations revealed no substantial changes. Tinnitus pitch was determined in both ears and ranged between 0.125 and 10 kHz. The pre-therapeutic mean tinnitus frequency was 4 kHz in vardenafil and 6 kHz in placebo subjects. At week 16 (LOCF), tinnitus pitch had remained stable in placebo patients, but increased in vardenafil patients to 6 kHz. However, there was a considerable inter-individual variability, even when the relative coefficient of variation was used (adjusted for sample size). Although descriptive statistics revealed different changes in both groups, exploratory ANCOVA did not demonstrate a significant vardenafil treatment effect.

The average tinnitus loudness remained relatively constant. Again, there was a considerable between-subject variability ranging from 2 to 110 dB SPL (sound pressure level). In the placebo group, the average loudness slightly increased in tinnitus-affected right ears (33 to 35 dB SPL) and remained stable in the left ears (38 dB SPL). Vardenafil-treated subjects showed a marginal increase by 1 dB SPL on average in right and left tinnitus ears (41 to 42 and 44 to 45 dB SPL, respectively).

**Figure 3:**
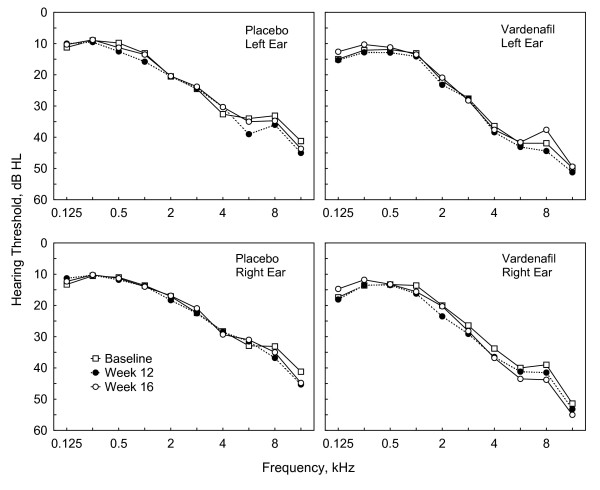
**Audiometric hearing thresholds from baseline to week 16 evaluated in left and right ears of vardenafil and placebo groups (ITT)**. For the sake of clarity, week 4 and LOCF data as well as whiskers are not shown.

As detected in the pure tone audiograms (0.125 to 10 kHz) of the patients, study medication did not result in any therapy-related impairment of hearing function. There was a pretherapeutic discrepancy between both treatment groups with the mean pantonal hearing loss being about 5 dB higher in the vardenafil group (28 dB) than in the placebo group (23 dB). However, the course of intra- and posttherapeutic values at all frequencies were comparable in verum and placebo patients without significant differences or trends. The particular hearing thresholds for both ears and treatment groups by visit (ITT) are displayed in Figure 3. The hearing thresholds did not show any significant changes.

#### Oxidative stress parameters in blood

The mean baseline values of MDA, TAS, PC and HCY are shown in Table 5. The mean baseline values were within the normal reference ranges and did not differ between both groups. The ANCOVA did not reveal any treatment effects in both groups from baseline to week 16 (LOCF) (ITT).

**Table 5:** Baseline values of oxidative stress-related parameters measured in blood of vardenafil- and placebo-treated patients (mean ± SD).

**Blood parameter**	**Vardenafil (N = 21)**	**Placebo (N = 21)**
Malondialdehyde (μmol/l)	0.07 ± 0.02	0.07 ± 0.02
Protein carbonyl (nmol/mg protein)	0.32 ± 0.12	0.29 ± 0.12
Total antioxidative status (mmol/l)	1.42 ± 0.30	1.45 ± 0.14
Homocysteine (μmol/l)	10.59 ± 3.52	9.27 ± 2.40

Four patients of the vardenafil group and 3 patients of the placebo group exposed HCY levels in serum (12–21 μmol/l) that are above the normal reference range regarding age and gender [43]. To exclude any influence of these high HCY values on tinnitus severity and hearing function, the whole statistics were repeated without the values of these 7 subjects. The analysis resulted in the same slight changes in the total score and subscores of the TQ as well as in the same audiometric findings like described above. ANCOVA did not reveal any treatment effects (data not shown).

## Discussion

Tinnitus is a complex symptom, whose specific pathophysiological connections and interactions are still not completely understood. Conditions like noise exposure, cardiovascular diseases, arterial hypertension, hyperlipoproteinemia, diabetes mellitus, or stress overload might cause hearing loss and tinnitus *via *changes of cochlear microcirculation with consecutive long-term impairment of blood circulation in the inner ear. Advances in methods of examining the cochlea and the human brain have increased specific knowledge about the origins of tinnitus [44,45]. Though, in many cases the actual cause remains unknown, somatic and psychosocial factors seem to be involved in its occurrence, centralization, chronification and possible decompensation. Due to this complexity, there is still no standard drug available for pharmacological treatment of "the" tinnitus. Our results demonstrate no difference in the treatment efficacy of chronic tinnitus between vardenafil and placebo. The primary efficacy criterion 'TQ total score' failed to demonstrate significant improvement compared to placebo in this study. This was also true for patients with high or low severity of tinnitus (dT, cT). Furthermore, the secondary efficacy analyses did not support the hypothesis that vardenafil could have beneficial therapeutic influence. Subjective reports of TQ subscales and general QoL areas (SF-36), objective audiometric examinations as well as investigated biomarkers for oxidative stress did not reveal any significant treatment effects.

Oxidative/nitrosative stress is recognized to be a prominent feature of many acute and chronic diseases and their progression [42]. In contrast, in the present study, none of the biomarkers of oxidative stress that were measured in the blood of patients with chronic tinnitus indicated statistically significant changes. Thus, the treatment of patients suffering from chronic tinnitus did not result in considerable modifications of the overall lipid and protein oxidation level, the total antioxidant capacity, or HCY concentration. HCY served as biomarker of oxidative stress in vascular tissue and is known to be associated with an increased risk of thrombosis and atherosclerosis [35]. However, exclusion of patients with high HCY values in serum offered consistent results without treatment effects or influence on tinnitus severity and hearing function. Regarding biomarker interpretation, some aspects have to be taken into account, e.g. different degrees of inter- and intraindividual variability and sensitivity, unknown amount of confounding and modifying factors, as well as uncertain specificity for different diseases. In patients with acute tinnitus, low NO levels were found in brain circulation reflux blood taken from the internal jugular vein, and a general cerebro-vascular endothelial dysfunction was discussed [46]. Furthermore, this study detected increased plasma levels of oxidative markers, which could not be confirmed in our patients with chronic tinnitus, where the blood was taken from the brachial veins. Therefore, global criteria of oxidative stress may not reflect specific oxidative stress in regions of the brain and inner ear.

Even if the investigated PDE5 inhibitor failed to show efficacy for treatment of chronic tinnitus, there is experimental evidence that the regional cochlear blood flow is actively regulated by the NO-system [20-22,47]. NO is produced by vascular endothelium and acts on the vascular smooth muscle to dilate the vessels and increase blood flow. In cochlear vasculature, NO plays a pivotal role in the regulation of vascular tone [21,22]. NO stimulates the soluble guanylate cyclase with subsequent cGMP formation, which activates protein kinases and leads to dephosphorylation of the myosin light chain [47]. Hence, drugs acting through the NO pathway should be highly capable of improving cochlear microcirculation. However, the mechanism of action of the PDE5 inhibitor used in the present study did not exert efficacy in respect to the mechanisms involved in chronic tinnitus. Thus, improving cochlear blood supply seems not be effective in the treatment of chronic inner ear disorders. The associated cochlear and central changes in chronic tinnitus are presumably already permanent and for that reason irreversible by NO. Conceivably, approaches for integration into acute tinnitus therapy could turn out to be more promising. Furthermore, various causes may represent the underlying pathophysiology of tinnitus. Sensorineural tinnitus is subdivided into motor, transduction, transformation, and extra-sensory tinnitus (type I-IV) [48]. Irregular release of neurotransmitters is believed to be involved in transformation tinnitus, whereas vascular disorders in the SV or cochlea may play a potential role in extra-sensory tinnitus [49]. Since diagnostics are still not available to precisely determine the patient's special subtype of sensorineural tinnitus, non-existence of vascular genesis obviously can not result in an adequate effect of vardenafil therapy.

Moreover, one might speculate that a 12-week period of drug application could have been too short or underdosed for treatment success. There is, however, evidence of efficacy for the long-term use of vardenafil 10 mg bid at 12 hr intervals [50] with sufficient therapeutic effect on ED [26,27]. The terminal half-life of vardenafil is approx. 4 hours. Therefore, the bi-daily regimen chosen was unlikely to cause cumulation. Since the pharmacodynamic effect of PDE5 inhibition is generally the same irrespective of the clinical indication, the selected dosage regimen exerted efficacy around the clock. Though, the missing effect of vardenafil on tinnitus symptoms is possibly due to the insufficient concentration of vardenafil in the inner ear. It is known that various medicaments applied systemically often fail to reach cochlear structures at adequate concentration and therefore, to achieve or increase therapeutic effects in the inner ear drugs have to be administered locally, for instance near or *via *the round window membrane [51,52].

With respect to safety of vardenafil in subjects diagnosed with tinnitus, no serious AEs were noticed. There were no deaths or clinically significant events like myocardial infarction or visual disturbances. Detected drug-related AEs were previously known from other studies or arise from the mechanism of vardenafil action. PDE5 inhibitors block the PDE5 induced degradation of cGMP [29] and thus lead to relaxation of smooth muscle cells lining the blood vessels, for instance those supplying the corpora cavernosa of the penis [53]. Conceivably, the increasing blood flow accounts for prolonged penile erection and nasal mucosal swelling in altogether 3 of our patients. All AEs were typical, generally transient, or disappeared after discontinuation of the study drug. Our AE profile was consistent with the safety profile in vardenafil studies on other indications. Prevalent drug-related AEs with an incidence of 2% or more are headache, flushing, rhinitis, dyspepsia, and dizziness [26,54]. Vardenafil given at 20 mg per day was shown to be safe for daily on-demand administration for longer periods of time [50].

Concerning our objective audiological investigations, the obtained results revealed no significant influence of vardenafil on average hearing thresholds or tinnitus parameters. However, mean tinnitus pitch remained stable in placebo patients, but slightly increased in verum subjects. Unfortunately, during our trial in 2007, there were first rare announcements of sudden decrease or loss of hearing in ED patients receiving PDE5 inhibitor therapy. Meanwhile, according to the US Food and Drug Administration at least 29 cases of such hearing impairment have occurred during post-marketing experience with all PDE5 inhibitors including vardenafil, with or without concomitant vestibular manifestations [55]. Hearing loss was unilateral in most cases, and temporary in about one-third of the patients. In one case, a 44-year-old man experienced permanent, bilateral sensorineural deafness 15 days after initiating therapy with the PDE5 inhibitor sildenafil; the patient did not have any prior or current risk factors for ototoxicity [56]. On the other hand, PDE5 inhibitors have been used worldwide so far by more than 40 million patients with ED and pulmonary arterial hypertension without otic side effects [23,27,28]. It is unclear whether these effects are directly related to PDE5 inhibitors or attributed to other factors (e.g., patient's underlying medical condition, concomitant use of other ototoxic drugs). However, a strong temporal relationship has been observed between the use of PDE5 inhibitors and the onset of hearing impairment in the reported cases. The 'precautions' and 'adverse reactions' sections of the approved product labeling had to be revised [50]. Due to these rare events, clinicians must advise patients about the possibility of hearing loss in case of intended vardenafil therapy. Patients should be instructed to discontinue PDE5 inhibitor treatment and seek medical attention immediately if sudden hearing loss occurs.

## Conclusion

In patients with chronic tinnitus, a 12-week trial with vardenafil tablets 10 mg bid was not superior over placebo. Our data did not show any significant treatment effects. The safety profile was favorable and consistent with that of other vardenafil studies. In spite of the particular role, which hypoxia and ischemia play in the pathogenesis of tinnitus, the PDE5-inhibitor-induced increase of cGMP and NO-dependent vasodilatation exerted no specific influence on tinnitus symptomatology. Considering our results and other studies with an unclear risk of rarely associated hearing impairment, systemic application of vardenafil or other PDE5 inhibitors prove to be inappropriate for therapy of chronic tinnitus. However, this is the only study of a PDE5 inhibitor that we are aware of having systematically assessed pure tone audiograms. This was performed in a double-blind manner versus placebo in a population of tinnitus patients. Given the above mentioned reports of sudden hearing loss in timely association with PDE5 inhibitors, it may be an important finding from this study that vardenafil did not cause statistically significant impairment of hearing thresholds in comparison with placebo in this population at risk according to the objective measures used.

## Abbreviations

AE(s): adverse event(s); AICA: anterior inferior cerebellar artery; ALT: alanine aminotransferase; ANCOVA: analysis of covariance; AST: aspartate aminotransferase; bid: twice a day; BMI: body-mass-index; cGMP: cyclic guanosine monophosphate; CK: creatine kinase; CK-MB: creatine kinase muscle-brain; cT: compensated tinnitus; dB: decibel; dT: decompensated tinnitus; ECG: electrocardiogram; ED: erectile dysfunction; EDTA: ethylendiamine tetra-acetic acid; ET-1: endothelin-1; hCG: human chorionic gonadotropine; HCY: homocysteine; HIF-1: hypoxia inducible factor 1; HL: hearing level; HPLC: high performance liquid chromatography; ITT: intent-to-treat; kHz: kilohertz; LOCF: last observation carried forward; LS-mean: least square mean; MDA: malondialdehyde; NO: nitric oxide; PC: protein carbonyl; PD: premature discontinuation; PDE5: phosphodiesterase type 5; PP: per-protocol; QoL: quality-of-life; SD: standard deviation; SF-36: Short Form 36 health survey; SMA: spiral modiolar artery; SPL: sound pressure level; SV: stria vascularis; TAS: total antioxidative status; TQ: tinnitus questionnaire; V1–5: visit 1–5.

## Competing interests

The authors declare that they have no competing interests.

## Authors' contributions

BM was responsible for conception and design of the project, interpretation of the data, drafting the manuscript. HH analyzed and interpreted the data, drafted and revised the manuscript. AJS interpreted the data and critically revised the manuscript. JS recruited the study participants, analysed and interpreted the audiometric data. JG collected, analyzed and interpreted the biomarker data. BFK collected, analyzed and interpreted the psychometric data. HK participated in study design, analysis and interpretation of laboratory data. UK participated in study coordination and interpretation of the data. TS interpreted the data and critically revised the manuscript. PPC analyzed and interpreted the data, drafted and critically revised the manuscript. All authors read and approved the final manuscript.
